# Annual transitions in nutrition, training, and interpersonal relationships of Japan's national champion university rowing team

**DOI:** 10.3389/fspor.2026.1808743

**Published:** 2026-06-10

**Authors:** Kimiyasu Hayakawa, Kazuki Mitsuyama, Tadashi Abe

**Affiliations:** 1 Faculty of Sports Science, Sendai University, Shibata, Japan; 2 Graduate School of Medicine, Tohoku University, Sendai, Japan

**Keywords:** annual transitions, elite athletes, nutrition behaviors, psychosocial factors, rowing performance, supplement knowledge, training practices, university rowing team

## Abstract

**Purpose:**

This study aimed to describe year-to-year transitions in nutrition-related behaviors, training practices, lifestyle indicators, and psychosocial factors among athletes in an elite Japanese university rowing program over a 6-year period (2018–2023). The objective was to characterize team-level seasonal patterns using a repeated cross-sectional approach.

**Methods:**

Anonymous questionnaires were administered annually after the competitive season (excluding 2021). All questionnaire items were assessed using either a 0–100 visual analogue scale (VAS) or open numerical responses. Descriptive statistics were calculated for each year and sex. Year-to-year differences were examined using one-way ANOVA, followed by Games–Howell *post hoc* tests to account for unequal variances and sample sizes. Effect sizes and 95% confidence intervals were reported. Because some athletes may have participated in multiple years, all analyses were interpreted within a repeated cross-sectional framework.

**Results:**

A total of 152 male and 59 female athletes participated across the five survey years. Several variables showed significant year-to-year differences, including body composition satisfaction, performance satisfaction, and training frequency, with patterns differing by sex. Female athletes exhibited markedly higher bodyweight and weight-training frequency in 2018 compared with later years. Many other variables—such as strength, power, endurance satisfaction, lifestyle regularity, supplement literacy, and psychosocial indicators—showed descriptive but non-significant fluctuations across seasons.

**Conclusion:**

This repeated cross-sectional analysis revealed substantial annual variation in nutritional behaviors, training exposure, lifestyle indicators, and psychosocial factors within a nationally competitive collegiate rowing program. The findings highlight the practical value of multidomain, team-level monitoring in environments characterized by high roster turnover, while emphasizing the descriptive and non-causal nature of the observed patterns.

## 1 Introduction

Athletic performance is shaped by a complex interplay of physical, nutritional, psychological, social, and lifestyle-related factors, as repeatedly demonstrated in previous research (1, 2). Domestic and international organizations have emphasized comprehensive performance enhancement strategies that integrate conditioning, nutrition, psychological support, sleep, and recovery (3, 4). In rowing, sport-specific conditioning and technical preparation are essential (5–7), and growing attention has been directed toward the influence of coach–athlete relationships and intrateam dynamics on performance environments (8, 9). Lifestyle behaviors—including sleep quality, daily routines, and overall lifestyle regularity—also contribute to physical readiness by interacting with training load and recovery capacity (10). These lifestyle-related factors represent fundamental components of athletes' preparedness and therefore warrant explicit consideration when examining seasonal transitions.

Dietary habits constitute another important component of performance readiness. Numerous studies have demonstrated associations between nutrition-related behaviors and physical attributes (11–13). In rowing, research has examined nutritional strategies surrounding training (14), energy balance (15, 16), hydration (17), and recovery-oriented interventions (18). However, empirical analyses of dietary habits among top-level rowers remain limited. A recent study by Mitsuyama et al. reported that higher-performing Japanese rowers were more likely to engage in intentional nutritional behaviors, such as checking ingredient labels, using supplements, and reducing fast-food intake (19). These findings highlight the potential relevance of nutrition-related literacy and habits in competitive rowing.

Contemporary sports science increasingly emphasizes integrated, multidomain perspectives rather than isolated evaluations of performance-related factors (20). University rowing programs, in particular, experience substantial annual roster turnover, making it difficult to track individual longitudinal changes but highly relevant to monitor team-level conditions across seasons. Understanding how nutritional behaviors, training practices, lifestyle regularity, and psychosocial relationships fluctuate annually may provide practical descriptive insights into seasonal team conditions and program development.

Based on this background, the present study aimed to describe year-to-year transitions in nutrition-related behaviors, training practices, lifestyle indicators, and psychosocial factors within an elite Japanese university rowing program, characterizing team-level seasonal patterns using a repeated cross-sectional approach.

## 2 Methods

### 2.1 Participants and study period

This study was conducted over a 6-year period from 2018 to 2023 and targeted the rowing team of S University, a team with a history of winning the All Japan University Rowing Championships. Participants included all male and female rowers from the first to fourth year, including those who did not compete in official races. Coxswains were excluded. All surveys were administered anonymously.

Given the organizational characteristics of university sports teams, in which team members change annually due to graduation and new enrollment, this study did not follow the same individuals over time. Instead, a repeated cross-sectional design was adopted to capture year-to-year team-level trends across multiple domains such as nutrition, training, and interpersonal relationships. Each academic year was analyzed as a separate annual sample for descriptive purposes; however, because some athletes may have participated in more than 1 year, full statistical independence across years cannot be assumed. Due to the anonymous data collection procedure, individual athletes could not be tracked longitudinally. The survey could not be conducted in 2021 due to COVID-19-related restrictions.

### 2.2 Survey content and procedures

#### 2.2.1 Survey items

Each year, immediately after the competitive season, athletes completed an anonymous questionnaire assessing multiple domains relevant to their experiences during the season. The questionnaire consisted of the following items:

- Satisfaction (VAS): body composition, athletic performance, muscular strength, power, endurance
- Training Practices: frequency of bodyweight strength training (days/week), frequency of weight training using equipment (days/week)
- Lifestyle Indicators: perceived regularity of daily routines (VAS), usual bedtime and wake-up time (hours and minutes)
- Supplement-Related Literacy: supplement knowledge (VAS), supplement understanding (VAS), daily frequency of protein powder intake (times/day)
- Interpersonal Relationships and Coaching Factors (VAS): relationships among team members, trust in teammates, perceived expectations from teammates, importance placed on coaches' instructional achievements, importance placed on coaches' athletic achievements, relationship with the head coach, trust in the head coach, perceived expectations from the head coach

All items were identical across survey years. To enhance methodological transparency, the full questionnaire used in this study is provided as Supplementary Table S1.

#### 2.2.2 Response format

All subjective evaluation items were assessed using a 0–100 visual analogue scale (VAS), whereas training frequency, protein intake, and sleep timing were recorded using open numerical responses. Because all questionnaire items were recorded as continuous numerical values (0–100 VAS scores or open numerical responses), all variables were eligible for descriptive and ANOVA-based analyses.

### 2.3 Statistical analysis

#### 2.3.1 Competition scoring system

Overall team rankings for each academic year were calculated according to the official scoring system of the All Japan University Rowing Championships. Points were awarded as follows: 1st = 5 points, 2nd = 3 points, 3rd = 2 points, 4th = 1 point. Total points across events determined the annual overall ranking for men and women.

#### 2.3.2 Analytical procedures

Because all questionnaire items were recorded as continuous values (VAS scores or numerical inputs), no categorical variables were included in the analyses, and all measures were treated as continuous outcomes in the ANOVA framework.

For all survey items, descriptive statistics (mean ± standard deviation) were calculated separately by sex and year. Year-to-year differences were examined using one-way analysis of variance (ANOVA). Because sample sizes differed across years and homogeneity of variance was not always satisfied (as assessed by Levene's test), *post hoc* comparisons were conducted using the Games–Howell method, which does not assume equal variances or equal sample sizes.

Statistical significance was defined as *α* = .05. Effect sizes were reported as eta-squared (*η*^2^) for ANOVA and Cohen's d for pairwise comparisons. Cohen's *d* values were calculated using pooled standard deviations adjusted for unequal variances and unequal sample sizes, consistent with the Games–Howell procedure. Ninety-five percent confidence intervals were calculated to describe the precision of observed differences.

Because multiple questionnaire items were analyzed, the analyses were considered exploratory, and *p*-values were interpreted cautiously. No formal correction for multiple testing was applied across all variables.

## 3 Results

A total of 152 male and 59 female athletes participated across the five survey years (2018, 2019, 2020, 2022, 2023). Year-specific descriptive statistics, ANOVA results, and Games–Howell *post hoc* comparisons are presented in Supplementary Tables S2-1 and S2-2 (male) and Supplementary Tables S3-1 and S3-2 (female).

### 3.1 Male athletes

#### 3.1.1 Variables showing significant year-to-year differences

##### 3.1.1.1 Body composition satisfaction

ANOVA indicated a significant main effect (*F* = 4.72, *p* = .001, *η*^2^ = .114). Games–Howell tests showed that:

- 2019, 2020, and 2023 had significantly higher VAS scores than 2022 (all *p* < .05).
- 2022 exhibited the lowest mean score (41.8 points).

##### 3.1.1.2 Performance satisfaction

A significant main effect was observed (*F* = 2.80, *p* = .028, *η*^2^ = .071). *Post hoc* tests identified:

- 2020 > 2022 (*p* = .028)

##### 3.1.1.3 Bodyweight training frequency

ANOVA showed a significant difference (*F* = 4.93, *p* < .001, *η*^2^ = .118). Games–Howell revealed:

- 2020 > 2018, 2019
- 2022 > 2018, 2019

##### 3.1.1.4 Weight training frequency

A significant main effect was found (*F* = 3.29, *p* = .013, *η*^2^ = .082). *Post hoc* tests showed:

- 2023 > 2019, 2020 (both *p* < .05)

##### 3.1.1.5 Interpersonal and coach-related variables

Significant ANOVA effects were observed for:

- relationships among members (*p* = .022)
- trust in members (*p* = .016)
- relationship with head coach (*p* = .032)
- trust in head coach (*p* = .014)

Games–Howell tests indicated that 2023 tended to show lower VAS scores than several earlier years, although only a limited number of pairwise contrasts reached significance.

### 3.2 Variables without significant differences (descriptive trends only)

The following variables did not show statistically significant year-to-year differences, but descriptive patterns were observed:

- Strength, power, and endurance satisfaction tended to increase toward 2023.
- Supplement knowledge and understanding increased gradually from 2018 to 2020.
- Daily routine quality showed no significant differences, although 2019 was descriptively lower.

These represent descriptive, non-significant fluctuations.

### 3.3 Female athletes

#### 3.3.1 Variables showing significant year-to-year differences

##### 3.3.1.1 Body composition satisfaction

ANOVA indicated a significant effect (*F* = 2.70, *p* = .040, *η*^2^ = .167). Games–Howell showed:

- 2019 > 2022 (*p* = .039)

##### 3.3.1.2 Performance satisfaction

A significant main effect was observed (*F* = 2.65, *p* = .043, *η*^2^ = .164). *Post hoc* tests identified:

- 2023 > 2022 (*p* = .031)

##### 3.3.1.3 Bodyweight training frequency

ANOVA showed a strong effect (*F* = 5.77, *p* < .001, *η*^2^ = .300). Games–Howell revealed:

- 2018 > 2019, 2020, 2022, 2023 (all *p* < .05)
- 2018 showed a markedly higher frequency (5.58 sessions/week).

##### 3.3.1.4 Weight training frequency

A significant effect was found (*F* = 3.68, *p* = .010, *η*^2^ = .214). *Post hoc* tests showed:

- 2018 > 2020 (*p* = .040)

#### 3.3.2 Variables without significant differences (descriptive trends only)

Although not statistically significant, the following descriptive patterns were observed:

- Supplement knowledge and understanding increased toward 2023.
- Strength, power, and endurance satisfaction tended to be higher in 2023.
- Daily routine quality was descriptively higher in 2018–2020 than in 2023.

These represent non-significant descriptive trends.

### 3.4 Competitive performance (descriptive context only)

For contextual reference, the men's overall team rankings at the All Japan University Rowing Championships were 2nd in 2018, 1st in 2019, 1st in 2020, 3rd in 2021, 15th in 2022, and 7th in 2023. The women's overall team rankings were 7th in 2018, 6th in 2019, 13th in 2020, 16th in 2021, 3rd in 2022, and 1st in 2023.

Because individual questionnaire responses were not statistically linked to team rankings, no inferential conclusions can be drawn regarding performance prediction.

Descriptively:

- Male championship years (2019–2020) occurred during seasons in which several physical and nutritional indicators showed higher VAS scores.
- The female high-performance year (2023) occurred during a season in which supplement literacy and performance satisfaction were descriptively higher.

These observations provide descriptive seasonal context only and should not be interpreted as evidence of performance-related associations.

## 4 Discussion

This study identified several statistically significant year-to-year differences in body composition satisfaction, performance satisfaction, and training frequency, whereas many other variables showed only descriptive fluctuations. These findings indicate that annual transitions in physical, nutritional, and psychosocial indicators were substantial, although these transitions should be interpreted strictly as descriptive patterns rather than indicators of performance-related outcomes.

This study examined 6-year transitions in nutritional behaviors, training practices, lifestyle indicators, and psychosocial factors within a nationally competitive university rowing program using a repeated cross-sectional design. After applying analytical procedures appropriate for unequal variances and unequal sample sizes—specifically the Games–Howell method—the results revealed several domains in which statistically robust year-to-year differences occurred, while many other patterns remained descriptive rather than inferential.

### 4.1 Male athletes

Among male athletes, significant year-to-year differences were observed in body composition satisfaction, performance satisfaction, bodyweight training frequency, weight training frequency, and several interpersonal or coach-related variables. The most pronounced finding was the markedly lower body composition satisfaction in 2022, which was significantly lower than in 2019, 2020, and 2023. These results indicate that perceived physical readiness varied across seasons. Performance satisfaction also differed significantly, with 2020 showing higher values than 2022.

Training-related indicators showed clear interannual variation. Bodyweight training frequency was significantly higher in 2020 and 2022 compared with 2018 and 2019, while weight training frequency was significantly higher in 2023 than in 2019 and 2020. These findings demonstrate that training exposure fluctuated across seasons, without implying any directional relationship with competitive outcomes.

Interpersonal and coach-related variables also showed significant differences, with 2023 tending to exhibit lower values than earlier years. Although not all pairwise contrasts reached significance, the overall pattern suggests that the psychosocial climate varied across seasons. Other variables—including strength, power, endurance satisfaction, supplement knowledge, supplement understanding, daily routine quality, and sleep duration—did not show statistically significant differences, although descriptive trends indicated gradual increases in supplement-related literacy and modest improvements in performance-related satisfaction toward 2023. These descriptive increases align with broader literature indicating that nutrition literacy can support informed decision-making among athletes (21–24). However, the present study did not evaluate the effectiveness of any educational interventions.

### 4.2 Female athletes

Among female athletes, significant year-to-year differences were observed in body composition satisfaction, performance satisfaction, bodyweight training frequency, and weight training frequency. Bodyweight training frequency showed the most pronounced pattern, with 2018 significantly exceeding all other years. Weight training frequency was also significantly higher in 2018 than in 2020. These findings indicate that training volume and modality varied across seasons. While gender-specific considerations are important in training and nutrition planning (25), the present results should be interpreted descriptively.

Body composition satisfaction was significantly higher in 2019 than in 2022, and performance satisfaction was significantly higher in 2023 than in 2022. These results indicate that perceived physical and performance readiness fluctuated across seasons, without implying any relationship with competitive outcomes (25–27).

Other variables—including strength, power, endurance satisfaction, lifestyle regularity, supplement knowledge, supplement understanding, and psychosocial indicators—did not show statistically significant differences. Descriptive patterns indicated higher performance-related satisfaction in 2023 and gradual increases in supplement literacy across years. These trends are consistent with previous reports on nutrition education in rowing populations (26, 28), although the present study did not assess intervention effects.

### 4.3 Integrated interpretation

Integrated interpretation in this manuscript refers to a descriptive synthesis of findings across physical, nutritional, lifestyle, and psychosocial domains. Across both sexes, the significant findings indicate that interannual variation occurred in specific domains—particularly body composition satisfaction and training frequency—while many other indicators fluctuated descriptively without reaching statistical significance.

Importantly, the descriptive patterns observed across seasons did not demonstrate any statistical associations with competitive outcomes. Although some seasons with higher competitive results occurred during years in which multiple indicators—such as physical satisfaction, training exposure, supplement literacy, and psychosocial climate—were descriptively higher, these observations represent contextual descriptions of seasonal conditions only. They should not be interpreted as evidence of relationships between questionnaire variables and performance outcomes.

This interpretation aligns with contemporary perspectives in sports science emphasizing the multifactorial and context-dependent nature of team environments. The present findings illustrate that team-level conditions in collegiate rowing fluctuate across seasons and that these fluctuations reflect complex patterns across multiple domains, without implying performance-related associations or causal pathways.

### 4.4 Methodological considerations

The use of the Games–Howell method strengthened the robustness of the statistical interpretation by appropriately addressing unequal variances and unequal sample sizes. This conservative approach ensured that only the most reliable year-to-year differences were identified as statistically significant. At the same time, the repeated cross-sectional design—necessitated by annual roster turnover—limits causal inference and precludes individual-level longitudinal interpretation.

### 4.5 Overall summary

Taken together, the updated analyses demonstrate that:

- Significant year-to-year differences were present in selected physical, training, and psychosocial indicators.
- Many variables showed only descriptive fluctuations, not statistical differences.
- Seasons with stronger competitive outcomes occurred during years in which multiple favorable indicators were descriptively higher, without implying any performance-related associations.
- Annual monitoring provides meaningful descriptive insights into team-level conditions in environments with high roster turnover.

These findings underscore the value of integrated, multifactorial monitoring frameworks in collegiate rowing programs and highlight the importance of considering physical, nutritional, lifestyle, and psychosocial factors collectively when evaluating seasonal transitions.

### 4.6 Limitations

This study has several limitations that should be considered when interpreting the findings. First, the repeated cross-sectional design—necessitated by annual roster turnover in collegiate sports—precludes the examination of individual longitudinal changes. As noted in the manuscript, “this study did not follow the same individuals over time… each academic year was treated as an independent cross-sectional dataset,” meaning that year-to-year differences cannot be interpreted as developmental trajectories or causal effects.

Second, all variables were assessed using self-reported questionnaires, including VAS-based subjective evaluations. Such measures are inherently susceptible to recall bias, social desirability bias, and individual differences in response style, which may have contributed to the substantial interannual variability observed in several indicators.

Third, the study was conducted within a single nationally competitive university rowing program. While this context provides a rare opportunity to examine multi-domain transitions in an elite collegiate environment, it also limits generalizability to other teams, competitive levels, or cultural settings.

Fourth, the absence of data in 2021 due to COVID-19 restrictions created a discontinuity in the temporal sequence, which may have influenced the interpretation of trends—particularly for variables that showed non-linear fluctuations across years.

Fifth, although the study included multiple domains—nutrition-related behaviors, training practices, lifestyle indicators, and psychosocial factors—no objective physiological or performance metrics (e.g., ergometer scores, body composition assessments, training load data) were collected. As a result, subjective perceptions could not be directly compared with objective indicators of physical readiness.

Finally, sample sizes varied across years, especially among male athletes, and homogeneity of variance was not always satisfied. Although the Games–Howell method was used to address unequal variances and unequal sample sizes, statistical power may have been insufficient to detect smaller effects, which may explain why many variables exhibited descriptive but non-significant fluctuations.

### 4.7 Practical applications

The findings of this 6-year repeated cross-sectional study provide descriptive information that may help coaches, support staff, and program directors understand seasonal team conditions in collegiate rowing environments characterized by annual roster turnover. Because only a limited number of variables showed statistically significant year-to-year differences—while many others fluctuated descriptively—the following points should be interpreted as contextual observations rather than recommendations or evidence of intervention effects.

#### 4.7.1 Monitoring indicators that showed statistically robust variation

Among male athletes, significant year-to-year differences were observed in body composition satisfaction, performance satisfaction, bodyweight training frequency, weight training frequency, and several interpersonal or coach-related variables. Among female athletes, significant differences were found in body composition satisfaction, performance satisfaction, and bodyweight and weighted training frequency.

These findings indicate that:

- Body composition satisfaction may function as a team-level indicator of perceived physical readiness across seasons.
- Training frequency, particularly bodyweight training among women, showed notable interannual variation that may warrant continued observation.
- Interpersonal and coach-related indicators among men, which were lower in 2023, may reflect shifts in psychosocial climate that could be monitored over time.

These points reflect descriptive patterns only.

#### 4.7.2 Using descriptive—but non-significant—patterns as contextual information

Although many variables did not reach statistical significance, several descriptive trends may help contextualize seasonal conditions:

- Supplement knowledge and understanding increased steadily in both sexes.
- Performance-related satisfaction (strength, power, endurance) tended to rise toward 2023, particularly among women.
- Daily routine quality fluctuated descriptively, including a lower value among men in 2019.

These observations provide contextual background and may inform future hypothesis generation.

#### 4.7.3 Emphasizing integrated, multidomain monitoring

A central descriptive finding was that seasons with stronger competitive outcomes occurred during years in which multiple indicators—such as physical satisfaction, training exposure, supplement literacy, and psychosocial climate—were simultaneously higher. This pattern highlights the potential value of monitoring multiple domains collectively rather than relying on isolated metrics. These observations do not imply performance-related associations.

#### 4.7.4 Practical considerations for collegiate rowing programs

Based on the descriptive patterns observed:

- Annual multidomain assessments may help identify seasonal shifts in team conditions.
- Opportunities for athletes to engage with nutrition-related information may support continued awareness of supplement use and dietary habits.
- Reviewing training load distribution—especially for female athletes—may help contextualize interannual variation in training exposure.
- Monitoring interpersonal and coach–athlete relationship indicators may help identify seasons in which psychosocial cohesion fluctuates.
- Lifestyle-related observations (sleep, recovery, daily structure) may help contextualize changes in routine quality across seasons.

These considerations are intended as descriptive insights rather than prescriptive recommendations.

#### 4.7.5 Value of repeated cross-sectional monitoring

Given the high roster turnover inherent in collegiate sports, repeated cross-sectional monitoring offers a feasible approach to:

- identify seasonal strengths and vulnerabilities
- contextualize year-to-year variation
- maintain continuity in program understanding despite changing personnel

These interpretations remain descriptive and non-causal.

## 5 Conclusions

This 6-year repeated cross-sectional study documented annual fluctuations in nutritional behaviors, training practices, lifestyle indicators, and psychosocial factors within a nationally competitive university rowing program. Several variables—particularly body composition satisfaction and training frequency—showed statistically robust year-to-year differences, whereas many others fluctuated descriptively without reaching statistical significance. These findings illustrate that team-level conditions in collegiate rowing vary across seasons and that such variation occurs across multiple domains.

The descriptive patterns observed in this study do not imply causal relationships or performance-related associations. Instead, they provide contextual information that may help characterize seasonal conditions in environments with substantial annual roster turnover. Repeated cross-sectional, multidomain monitoring appears to be a feasible approach for documenting these transitions over time. The interpretations presented here remain descriptive and non-causal, offering contextual insight rather than evidence supporting specific interventions or performance outcomes (29–32).

## Data Availability

The raw data supporting the conclusions of this article will be made available by the authors, without undue reservation.
