# Supplementary material for: Annual transitions in nutrition, training, and interpersonal relationships of Japan's national champion university rowing team
Source: Front Sports Act Living. 2026 Jun 10;8:1808743. doi: 10.3389/fspor.2026.1808743 (PMC13291930; doi:10.3389/fspor.2026.1808743)

**Table 1. Survey questionnaire used in this study.**

This table presents the full set of questionnaire items administered annually to all athletes. Subjective evaluations were assessed using a 0–100 visual analogue scale (VAS), whereas training frequency, protein intake, and sleep timing were recorded using numerical responses. All items were identical across survey years and were designed to capture multi‑domain indicators related to body composition satisfaction, performance satisfaction, training practices, lifestyle regularity, supplement‑related literacy, and psychosocial or coach‑related factors.

1. 【Body Composition Satisfaction(VAS)】Were you satisfied with your body composition (body weight, body fat percentage, muscle mass, etc.) during this season?


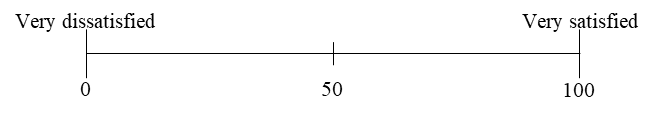


2. 【Performance Satisfaction(VAS)】Were you satisfied with your athletic performance during this season?


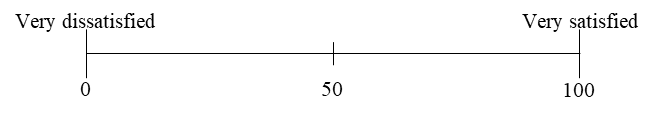


3. 【Bodyweight Training Frequency (in-season)】Number of days you performed bodyweight strength training during the competition season:

　　approximately (  ) days per week

4. 【Weight Training Frequency (in-season)】Number of days you performed weighted strength training (e.g., using barbells, dumbbells, or machines) during the competition season:

　　approximately (  ) days per week

5. 【Strength Satisfaction(VAS)】Were you satisfied with your muscular strength (e.g., maximum barbell lifting capacity) during this season?


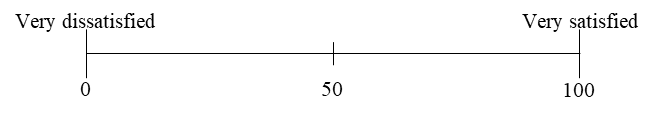


6. 【Power Satisfaction(VAS)】Were you satisfied with your power (i.e., the ability to exert muscular force instantaneously: force × velocity) during this season?


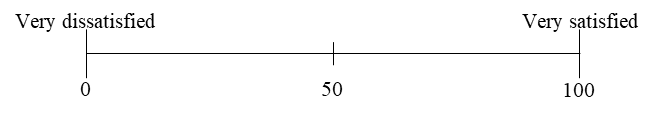


7. 【Endurance Satisfaction(VAS)】Were you satisfied with your endurance during this season?


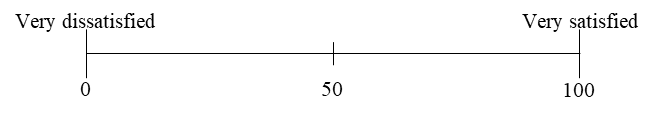


8. 【Daily Routine Quality(VAS)】Is your daily routine—from waking up to going to bed—well regulated?


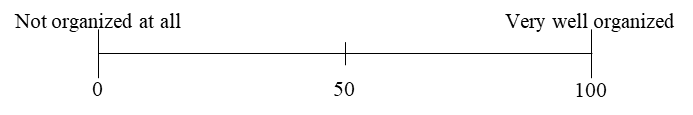


9. 【Sleep Duration (minutes)】

9-1. Please tell us your approximate usual bedtime.

　　　(  ) : (  ) (24-hour format)

9-2. Please tell us your approximate usual wake-up time.

　　　(  ) : (  ) (24-hour format)

10. 【Supplement Knowledge(VAS)】Do you think you have knowledge about supplements?


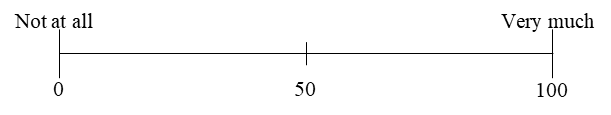


11. 【Supplement Understanding(VAS)】Do you think you are able to understand supplements?


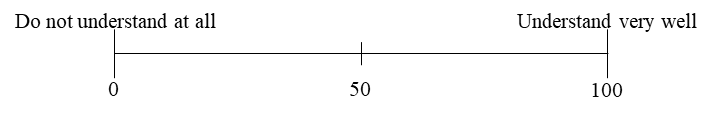


12. 【Protein Intake Frequency (per day)】How many times per day do you consume powdered protein?

　　　(  ) times

13. 【Relationships Among Members(VAS)】Are your relationships with other team members good?


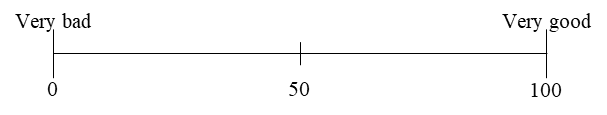


14. 【Trust in Members(VAS)】Do you trust the other team members?


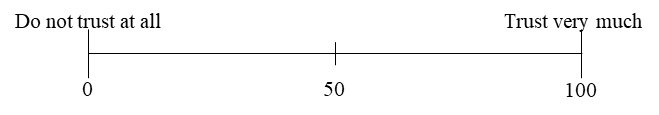


15. 【Being Trusted by Members(VAS)】Do you feel that other team members have expectations of you?


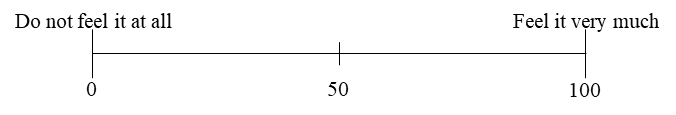


16. 【Importance of Coach’s Instructional Achievements(VAS)】Do you place importance on the coaching achievements of your coaches (e.g., head coach, assistant coaches)?


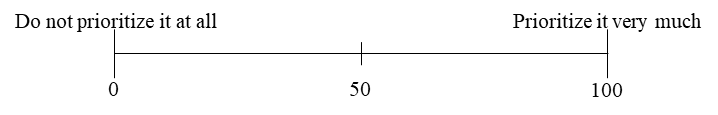


17. 【Importance of Coach’s Athletic Achievements(VAS)】Do you place importance on the competitive achievements that your coaches (e.g., head coach, assistant coaches) accomplished during their own athletic careers?


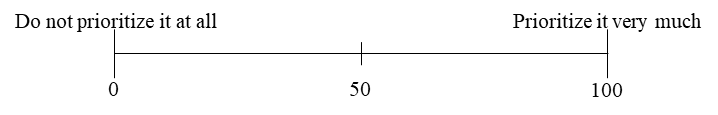


18. 【Relationship with Head Coach(VAS)】Is your relationship with the head coach good?


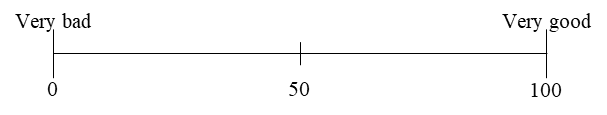


19. 【Trust in Head Coach(VAS)】Do you trust the head coach?


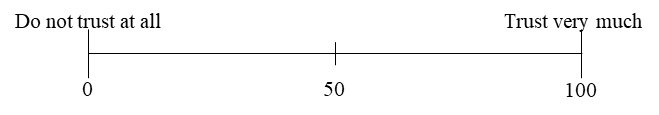


20. 【Expectations from Head Coach(VAS)】Do you feel that the head coach has expectations of you?


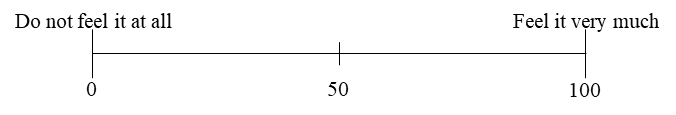

Supplement: Supplementary file 1 [file Supplementaryfile1.docx]
